# Contraceptive-induced amenorrhoea leads to reduced migraine frequency in women with menstrual migraine without aura

**DOI:** 10.1186/1129-2377-15-30

**Published:** 2014-05-17

**Authors:** Kjersti Grøtta Vetvik, E Anne MacGregor, Christofer Lundqvist, Michael Bjørn Russell

**Affiliations:** 1 Head and Neck Research Group, Research Centre, Akershus University hospital, 1478, Lørenskog, Norway; 2 Institute of Clinical Medicine, Campus Akershus University Hospital, University of Oslo, Oslo, Norway; 3 Centre for Neuroscience and Trauma, Blizard Institute, Barts and the London School of Medicine and Dentistry, London, UK; 4 HØKH, Research Centre, Akershus University Hospital, 1478, Lørenskog, Norway; 5 Department of Neurology, Akershus University Hospital, 1478, Lørenskog, Norway

**Keywords:** Menstrual migraine without aura, Migraine, Amenorrhoea, Contraception, Hormones, Estrogen, Progestin, Prostaglandins, Ovulation

## Abstract

**Background:**

Menstrual migraine without aura (MM) affects approximately 20% of female migraineurs in the general population. The aim of the present study was to investigate the influence of contraception on the attacks of migraine without aura (MO) in women with MM.

**Findings:**

141 women from the general population with a history of MM according to the International Classification of Headache Disorders II (ICHD II) were interviewed by a headache specialist. Of 49 women with a history of MM currently using hormonal contraception, 23 reported amenorrhoea. Significantly more women with amenorrhoea reported no MO- days during the preceding month compared to women without amenorrhoea (OR 16.1; 95% confidence interval (CI) 1.8-140.4; *P* = 0.003). A reduction of MO-frequency was more often reported in women with than without amenorrhoea (OR 3.5; 95% CI 1.1-11.4; *P* = 0.04).

**Conclusion:**

Amenorrhoea leads to a reduction of MO-frequency in women with MM using hormonal contraceptives. Future prospective studies on MM should focus on contraceptive methods that achieve amenorrhoea.

## Findings

### Introduction

Menstrual migraine without aura (MM) affects approximately 20% of female migraineurs in the general population [1,2]. The appendix criteria of the International Classification of Headache Disorders (ICHD) II and III beta version defines MM as attacks of migraine without aura (MO) which occur on day 1 ± 2 (i.e. days −2 to +3) of menstruation in at least 2/3 menstrual cycles [3,4].

The pathophysiology of MM is not fully understood and probably heterogeneous. Clinical observations indicate an increased incidence of MO in conditions with falling levels of plasma estrogen such as in the perimenstrual phase of the menstrual cycle [5], the hormone-free interval in women taking combined hormonal contraception (CHC) [6], and in the days directly following a childbirth [7]. Experimental studies have shown that MM can be prevented by supplementing estrogen at the time of the natural decline during the late luteal phase of the cycle [8]. This led to the hypothesis that MM is triggered by the decline in plasma estrogen during the late luteal phase of the cycle following a period of oestrogen ‘priming’ [9]. Perimenstrual prostaglandin may also play a direct or indirect role in triggering MM attacks [10].

Anecdotally, MM is more likely to improve in women who achieve amenorrhoea [11], but data regarding this possible effect are lacking.

The aim of our study was to explore the influence of amenorrhoea on all MO attacks in women with MM using hormonal contraceptive methods.

### Material and method

A random sample of 5000 women from the general population aged 30–34 years received a mailed screening questionnaire about their migraine and the relationship of attacks to menstruation. Women with self-reported migraine in at least 50% of menstruations and <180 headache days/year were invited to participate in a semi-structured headache interview six years later. At that time the participants were aged 36–40 years. The interview included questions about migraine, menstruation and contraception. All women were asked about type of current hormonal contraception, duration of use and whether amenorrhoea had arisen after initiating the method. Additionally, a subgroup analysis of contraceptive methods which are expected to inhibit ovulation (i.e. CHC, desogestrel-only pill and depot medroxyprogesterone acetate injection) and methods which are expected not to inhibit ovulation (i.e. LNG-IUS and progestin-only mini-pill) was made. Headaches were classified according to the ICHD II-criteria, including the appendix criteria for menstrual migraine (i.e. A 1.1.1 pure menstrual migraine and A 1.1.2 menstrually-related migraine) [4]. In the current paper, the term menstrual migraine (MM) covers both A1.1.1 and A 1.1.2. Amenorrhoea was defined as the absence of menses for three months in a woman with previously normal menstruation [12].

The following question was evaluated: “Did your MO change after onset of current contraceptive method?” with respect to frequency, intensity and duration. This question referred to all MO-attacks, not exclusively MM-attacks, and responses were categorized as; fewer/less severe/shorter, no change, more frequent/more severe/longer duration. In addition, women were asked to state the number of migraine days per month on average over the last three months preceding the interview.

The interviews were conducted by a neurologist with special interest in headache disorders (KGV). A more exhaustive description of the method has been published elsewhere [2].

#### Statistics

The data were analyzed using Statistical Package of Social Sciences (SPSS) version 20. A 2-sided Fisher’s exact test mid-p variant was used to compare two groups with categorical variables. Differences regarding self-reported changes of MO-frequency are presented as odds ratio (OR) with 95% confidence intervals. Due to skewed distribution and low number of participants, non-parametric tests (Independent samples median test) were used to calculate differences in continuous variables. A p-level <0.05 was considered significant.

#### Ethics

The study was approved by the Norwegian Regional Committee for Medical Research Ethics and the Norwegian Data Protection Authorities. The participants received written and verbal information about the project and inclusion was based on informed consent.

### Results

**Figure 1:**
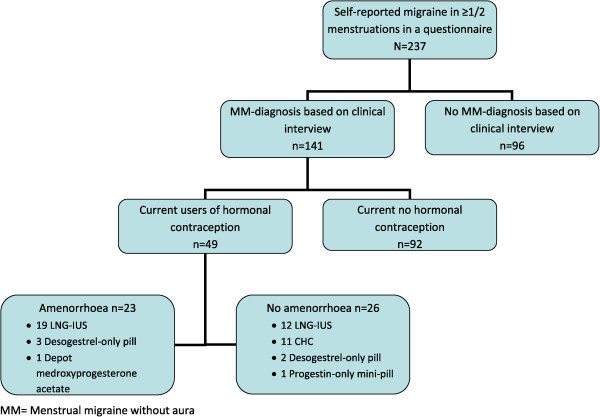
**Flow chart of the study.** LNG-IUS = Levonorgestrel interuterine system. CHC = combined hormonal contraception.

The questionnaire response rate was 73%. Of 360 women meeting the inclusion criteria, four had emigrated, nine had insufficient language skills, 39 did not reply to five or more phone calls and 71 declined to participate due to lack of time, no interest or acute illness. Of the remaining 237 women interviewed, 141 women were diagnosed with MM. Forty-nine women were current users of hormonal contraception, of whom 23 reported amenorrhoea (Figure 1). Women with amenorrhoea had used current contraceptive method for 10–132 months and significantly longer than women without amenorrhoea (Table 1). Migraine prophylaxis was used by four women, of whom one had amenorrhoea (Table 1).

**Table 1:** Population variables

	**Amenorrhoea (n = 23)**	**Menstruation/withdrawal bleeding (n = 26)**	**p-value**
**Continuous variables**^**1**^	*Median*		
Age at migraine onset	18	18	0.92
Age at menstrual migraine onset	25	22	0.48
Duration of hormonal contraceptive use (months)	48	24	0.02
**Dichotomous variables**^**2**^	*n (%)*		
Migraine prophylaxis, n (%)	1 (4)	3 (12)	0.42
Triptan use, n (%)	15 (65)	14 (54)	0.44
Ever consulted a neurologist for migraine, n (%)	8 (35)	8 (31)	0.78
Endometriosis	2 (9)	0 (0)	0.22

^1^Independent samples median test. ^2^Fischers exact mid-p test.

Significantly more women with amenorrhoea reported a reduction of MO-frequency after initiation of current contraceptive method than women with menstrual/withdrawal bleeds (OR 3.5, 1.1-11.4, P = 0.04). Intensity and duration was not significantly different in the two subgroups (Table 2). Only 1/23 woman with amenorrhoea reported increased frequency, intensity and duration of MO-attacks, while 3/26 women without amenorrhoea reported increased frequency and 2/26 longer duration and higher pain intensity. The remaining reported no change.

**Table 2:** Effects of amenorrhoea on migraine without aura (MO) in women with menstrual migraine (MM)

	**Amenorrhoea (N = 23)**	**Menstruation/withdrawal bleeding (N = 26)**			
***Proportions with reduced***	***% (n)***	***% (n)***	***OR***	***95% CI***	***p***
Attack frequency	61 (14)	31 (8)	3.5	1.1-11.4	0.04
Attack intensity	30 (7)	19 (5)	1.8	0.5-6.9	0.39
Attack duration	30 (7)	23 (6)	1.5	0.4-5.2	0.58
*Proportion of women reporting no MO-days during the preceding month*	39 (9)	4 (1)	16.1	1.8-140.4	0.003

A larger proportion of amenorrhoeic women reported no MO-days during the preceding month than those with menstrual/withdrawal bleeds (OR 16.1, 1.8-140.4, P = 0.003).

A subgroup analysis of methods with and without expected inhibition of ovulation showed that reduced frequency of MO was less often reported in women using expected anovulatory contraceptive methods than in women using methods with expected preserved ovulation (OR 0.2, 0.1-0.9, P = 0.03). Intensity and duration of MO-attacks were not different between the two subgroups.

### Discussion

In this study, achievement of amenorrhoea was associated with a higher proportion of self-reported reduction of MO-frequency in women with MM compared to women who were not amenorrhoeic.

Based on the hypothesis that estrogen withdrawal triggers MM attacks, the focus in previous studies on MM has been to avoid this estrogen drop and little attention has been directed toward other potential mechanisms [13,8,14].

CHCs contain a combination of estrogens and a progestin most of which have a standard formulation of 21 days active hormones followed by a 7-day hormone-free interval. The contraceptive effect of CHCs is primarily from suppression of ovulation. During the hormone-free interval, usually associated with withdrawal bleedings, migraine is more likely to occur. This has been explained by the decline in estrogen during this period. Extended cycle regimens of CHC, such as formulations with 84 days active hormones before a 7-day hormone-free interval, are associated with reduced incidence of MM attacks, an effect which has been explained by stabilization of estrogens throughout the cycle [14]. Notably, these regimens are also associated with reduced incidence of withdrawal bleeds. It is unknown whether it is the stabilization of estrogens or achievement of amenorrhoea – or both, that are the actual mechanisms of action. A shortened hormone-free interval, from a 21/7- to a 24/4-regimen, in women taking CHC showed a positive effect on MM with significant reduction of attack duration and pain intensity. This effect was explained by a shorter period of hypoestrogenism in the 24/4-regimen [15]. Differences in bleeding profiles between the groups were not reported.

Progestin-only methods provide contraceptive effects in different fields, depending on the doses and route of administration. Ovulation is usually inhibited by high doses, while lower doses act by thickening of cervical mucus and endometrial suppression [16]. Estrogen-withdrawal is also prevented with some progestin-only methods, provided that ovarian activity is sufficiently suppressed. The effect of continuous oral progestin, given in doses high enough to inhibit ovulation, was evaluated in a small sample of female migraineurs of whom 13/22 reported MM [11]. Only 5/13 women with MM had a ≥50% reduction in migraine frequency. Unscheduled bleeding was reported by 14/22 of all women and among these, 9 suffered migraine during such bleeding. The occurrence of breakthrough bleeding was considered to result from incomplete suppression of ovarian activity resulting in fluctuating estrogen levels, which can occur even when ovulation is inhibited. This failure to achieve amenorrhoea, may explain the “disappointing” results. The two patients who achieved amenorrhoea, likely to be associated with greater ovarian suppression, became headache free [11].

On this basis, it could be hypothesized that MM will persist in women using contraceptive methods that result in estrogen withdrawal as a consequence of the endogenous ovarian cycle (e.g. LNG-IUS) or withdrawal of exogenous estrogen (CHC). Further, progestin-only methods that inhibit ovulation (e.g. desogestrel pill), should be associated with improvement in MM.

However, we found that the benefit of amenorrhoea was independent of the effect of the method on ovulation. Of the amenorrhoeic group, 19/23 were using the LNG-IUS. The plasma levels of progesterone and estrogens are similar in women with and without amenorrhoea using the LNG-IUS. The incidence of ovulation is also similar in both groups [17]. This indicates that absence of menstrual bleeding is not a predictor of ovarian function but a result of the local effect within the endometrial cavity with strong suppression of growth and induction of endometrial insensitivity to ovarian estradiol; only about 15% of women using LNG-IUS develop anovulation [17].

Of the group who were not amenorrhoeic, 24/26 were using methods associated with continuing estrogen withdrawal (LNG-IUS = 12, CHC = 11, progestin-only ‘mini-pill’ = 1). Hence the benefit of amenorrhoea appears to be independent of the effect of the method on estrogen-withdrawal. A possible explanation may be the oligo-amenorrhoea that follows 6–12 months after insertion of LNG-IUS and consequent effect on prostaglandins, which have been implicated in MM, particularly associated with dysmenorrhoea [18]; a reduction in menstrual flow is associated with reduced levels of prostaglandins reaching the systemic circulation in the early menstrual phase, potentially reducing the effect on migraine.

#### Strengths and limitations

This is one of few studies exploring the effect of progestin-induced amenorrhoea in menstrual migraineurs. The women were diagnosed by a neurologist after a clinical interview according to the ICHD II-criteria, but the diagnosis was not confirmed by prospective headache diaries. The retrospective design is subject to recall bias, but since many women reported amenorrhoea at time of interview, it was not possible to confirm the MM-diagnosis by prospective diaries. The MM-diagnoses are consequently based on symptoms experienced before achievement of amenorrhoea.

Women with amenorrhoea had used the contraceptive method significantly longer than women without amenorrhoea. This may be explained by satisfaction with the method, but other reasons such as wish to avoid pregnancy or co-morbid menstrual disorders could also explain this difference. Our results may have been influenced by changes in medication. However, migraine prophylaxis was used by more women with menstruation/withdrawal bleeds than amenorrhoeic women.

We made a subgroup analysis of methods with/without inhibition of ovulation. The effect of each contraceptive method was based on the known mechanism of action in the majority of women, but it is acknowledged that ovulation can occur in some women using oral desogestrel and ovulation is inhibited in some women using the LNG-IUS, irrespective of amenorrhoea.

Due to the relatively small number of women with MM using hormonal contraception and the retrospective design of our study, the results should be interpreted with some caution and need confirmation by a larger prospective study.

### Conclusion and the future

Women with MM benefited from using contraceptive methods leading to amenorrhoea, while methods with expected anovulation had no such effect. These findings suggest that neither inhibition of ovulation nor prevention of estrogen withdrawal are necessary for the management of MM, and that more focus should be given to amenorrhoea. Future studies should also explore the underlying pathophysiology.

## Abbreviations

MO: Migraine without aura; MM: Menstrual migraine without aura; ICHD: The International Classification of Headache Disorders; LNG-IUS: Levonorgestrel intrauterine device; CHC: Combined hormonal contraception.

## Competing interests

The authors declare that they have no competing interests.

## Authors’ contributions

MBR had the original idea for the project. MBR, KGV, EAM and CL contributed to design of the study. EAM had the idea for the analyses of hormonal contraception in this manuscript. KGV performed all interviews and drafted the manuscript. MBR, EAM and CL reviewed the first and subsequent drafts. All authors read and approved the final manuscript.
